# Evaluation of a tumor electric field treatment system in a rat model of glioma

**DOI:** 10.1111/cns.13441

**Published:** 2020-07-30

**Authors:** Hao Wu, Chenxi Wang, Jialin Liu, Dan Zhou, Dikang Chen, Zhixiong Liu, Anhua Wu, Lin Yang, Jiusheng Chang, Chengke Luo, Wen Cheng, Shuai Shen, Yunjuan Bai, Xuetao Mu, Chong Li, Zhifei Wang, Ling Chen

**Affiliations:** ^1^ Chinese PLA Institute of Neurosurgery Chinese PLA General Hospital and PLA Medical College Beijing China; ^2^ National Institutes for Food and Drug Control Beijing China; ^3^ Hunan An Tai Kang Cheng Biotechnology Co., Ltd Changsha China; ^4^ Xiangya Hospital Central South University Changsha China; ^5^ The First Hospital of China Medical University Shenyang China; ^6^ Chinese Academy of Military Sciences Beijing China; ^7^ The Third Medical Center of The General Hospital of PLA Beijing China; ^8^ Institute of Biophysics Chinese Academy of Sciences Beijing China; ^9^ The Third Xiangya Hospital of Central South University Changsha China

**Keywords:** cell death, electric field therapy, glioma, survival, tumor electric field treatment system, tumor size

## Abstract

**Objective:**

Glioma is a devastating disease lacking effective treatment. Tumor electric field therapy is emerging as a novel non‐invasive therapy. The current study evaluates the efficacy and safety of a self‐designed tumor electric field therapy system (TEFTS ASCLU‐300) in a rat orthotopic transplantation model of glioma.

**Methods:**

A model of intracranial orthotopic transplantation was established in rats using glioma C6 cells. For electric field therapy, glioma‐bearing rats were exposed to alternating electric fields generated by a self‐developed TEFTS starting on either 1st (Group 2) or 3rd (Group 3) day after transplantation, while other conditions were maintained the same as non‐treated rats (Group 1). Glioma size, body weight, and overall survival (OS) were compared between groups. Immunohistochemical staining was applied to access tumor cell death and microvessel density within the tumor. In addition, the systemic effects of TEFTS on blood cells, vital organs, and hepatorenal functions were evaluated.

**Results:**

TEFTS treatment significantly elongated the OS of tumor‐bearing rats compared with non‐treated rats (non‐treated vs treated: 24.77 ± 7.08 days vs 40.31 ± 19.11 days, *P* = .0031). Continuous TEFTS treatment starting on 1st or 3rd day significantly reduced glioma size at 2 and 3 weeks after tumor cell inoculation (Week 2: Group 1:289.95 ± 101.69 mm^3^; Group 2:70.45 ± 17.79 mm^3^; Group 3:73.88 ± 33.21 mm^3^, *P* < .0001. Week 3: Group 1:544.096 ± 78.53 mm^3^; Group 2:187.58 ± 78.44 mm^3^; Group 3:167.14 ± 109.96 mm^3^, *P* = .0005). Continuous treatment for more than 4 weeks inhibited tumor growth. The TEFTS treatment promoted tumor cell death, as demonstrated by increased number of Caspase 3^+^ cells within the tumor (non‐treated vs treated: 38.06 ± 10.04 vs 68.57 ± 8.09 cells/field, *P* = .0007), but had minimal effect on microvessel density, as shown by CD31 expression (non‐treated vs treated: 1.63 ± 0.09 vs 1.57 ± 0.13% of positively stained areas, *P* > .05). No remarkable differences were observed in hepatorenal function, blood cell counts, or other vital organs between non‐treated and treated groups.

**Conclusion:**

The TEFTS developed by our research team was proved to be effective and safe to inhibit tumor growth and improve general outcomes in a rat model of brain glioma.

## Graphical abstract

The self‐designed TEFTS was proved to be effective and safe to inhibit tumor growth and improve general outcomes of Glioma.The instrument with random sequential electric field direction and adjustable intensity and frequency can apply electric field to the rapidly proliferating tumor cells from all directions in order to achieve the best curative effects.

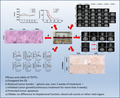

## 1 INTRODUCTION

Glioma is the most prevalent type of brain tumor in China and in the United States,^1^, ^2^ accounting for about 80% of malignant intracranial tumors. Its high morbidity, mortality, recurrence rate, and poor 5‐year survival rate make it a serious medical problem that is difficult to tackle. Many traditional therapies and the emerging immune therapies that showed promising effects on other solid tumors have failed to slow down the progress of glioma.^3^, ^4^ There is an increased need for alternative therapies for this devastating disease.

Tumor electric field therapy or Tumor Treating Fields (TTFields) was implemented as a novel non‐invasive physiotherapy in the early 2000. TTFields are alternating electric fields of intermediate frequency ranging between 100‐300 kHz and typically 1‐3 V/cm intensities. TTFields generators have been shown in a variety of in vitro and in vivo systems to exert antimitotic effects on dividing cells.^4^, ^5^, ^6^, ^7^ Accumulating studies reveal that TTFields may inhibit tumor growth through multifaceted mechanisms. First, the electric fields are able to interfere cancer cell division by disrupting the assembly of mitotic spindles in dividing cancer cells during metaphase and anaphase. Second, the non‐uniform electric fields result in membrane blebbing and multinucleation during telophase, which lead to the generation of abnormal daughter cells and induction of cell death.^4^, ^6^, ^7^ Finally, the electric fields therapy is recently reported to work through a cell line‐specific mechanism to stimulate Ca^2+^entry via Cav1.2, which culminates in cell cycle arrest, DNA degradation, and cell death.^8^ Intriguingly, the TTfields preferentially target tumor cells but leave the normal cells intact, rendering it a promising therapy for cancers. A recent clinical trial report confirmed the efficacy and safety of electric field therapy using Novocure TTFields, when applied in conjugation with temozolomide, in Asian patients with newly diagnosed glioblastoma patients.^9^ In addition, TTFields have been tested in clinical trials in patients with other solid tumors outside the brain.^10^ In 2017, the FDA has designated the TTFields delivery system as a treatment for pleural mesothelioma.

Our research team has designed and manufactured a Tumor Electric Field Treatment System (TEFTS ASCLU‐300) with the intention to utilizing alternating electric fields for tumor treatment. Here, we validate the safety and efficacy of this instrument in a rat orthotopic transplantation model of glioma. TEFTS might be used as a clinical feasible therapy for human glioma.

## 2 MATERIALS AND METHODS

### 2.1 Animals

**FIGURE 1:**
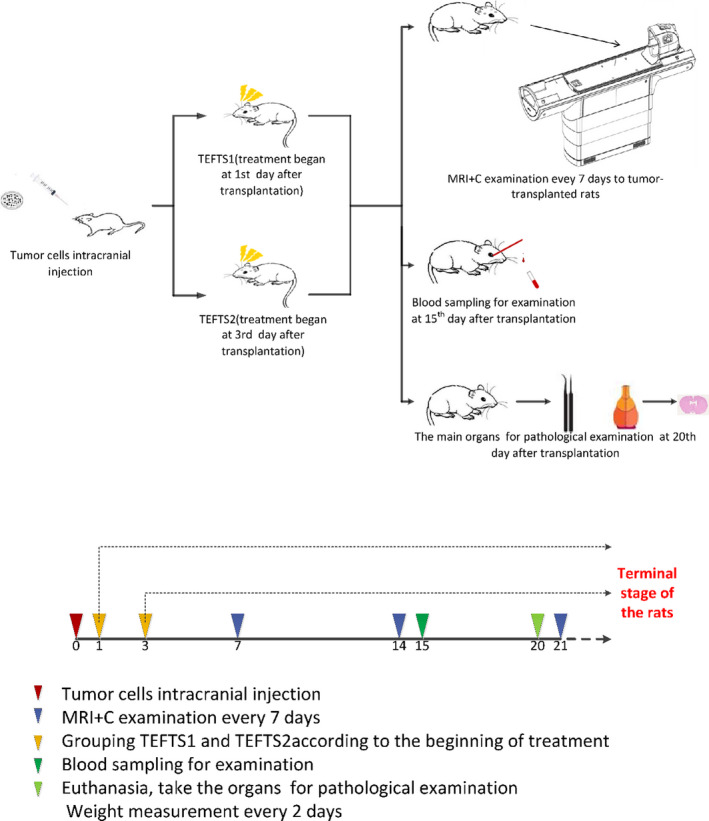
Flow chart of experimental design and main endpoint measurements

Male CD®(Sprague Dawley) IGS rats (6‐8‐week‐old) were purchased from Beijing Vital River Laboratory Animal Technology Co. Ltd. All rats were kept and fed in the laboratory animal center of the Institute of Biophysics, Chinese Academy of Sciences in Beijing. Blood was collected on day 15 after TEFTS treatment for hepatic and renal function test and blood test. Liver, kidney, and brain tissues were collected at day 20 after treatment for HE staining and pathological examination. All animal handling, surveillance, and experimentations were performed in strict accordance with the rules of animal experimental ethics by the Animal Care and Use Committee of Chinese PLA General Hospital. The animal experimental design is illustrated in Figure 1.

### 2.2 Cell culture

The rat C6 glioma cell line was purchased from the Institute of Basic Medicine, Chinese Academy of Medical Sciences. Cells were cultured in DMEM medium with high glucose (Dulbeccos' Modified Eagle's Medium‐High Glucose, Thermo Scientific Fisher) supplemented by 10% V/V FBS (Fetal Bovine Serum, Thermo Scientific Fisher) and incubated at 37°C and 5% CO_2_. Cells at exponential growth stage were released by 0.25% trypsin and adjusted to 5 × 10^7^ cells/mL in PBS for use.

### 2.3 Establishment of glioma orthotopic transplantation model in rats

Animals were anesthetized with isoflurane inhalation and fixed on a stereotactic frame. Animals were maintained at 36.5‐37.5°C with a heating pad. After sterilization, an incision was made approximately 1 cm from the sagittal line. A small burr hole (diameter of 1 mm) was drilled by a dental drill on the right frontal bone. A 26‐G Hamilton syringe was used to inject 10 μL C6 glioma cell suspension (containing 5 × 10^5^ cells) into the caudate nucleus according to the following coordinates: 1 mm anterior, 3 mm lateral to the bregma, and 6 mm below the skull (with a 1 mm withdrawal later). The cell suspension was slowly injected at a rate of 1 μL/min. The needle was maintained for 5 minutes after injection and retracted at 2 mm/min. The burr hole was sealed with sterilized medical bone wax, and the skin was sutured.

### 2.4 Electrode setting and installation

**FIGURE 2:**
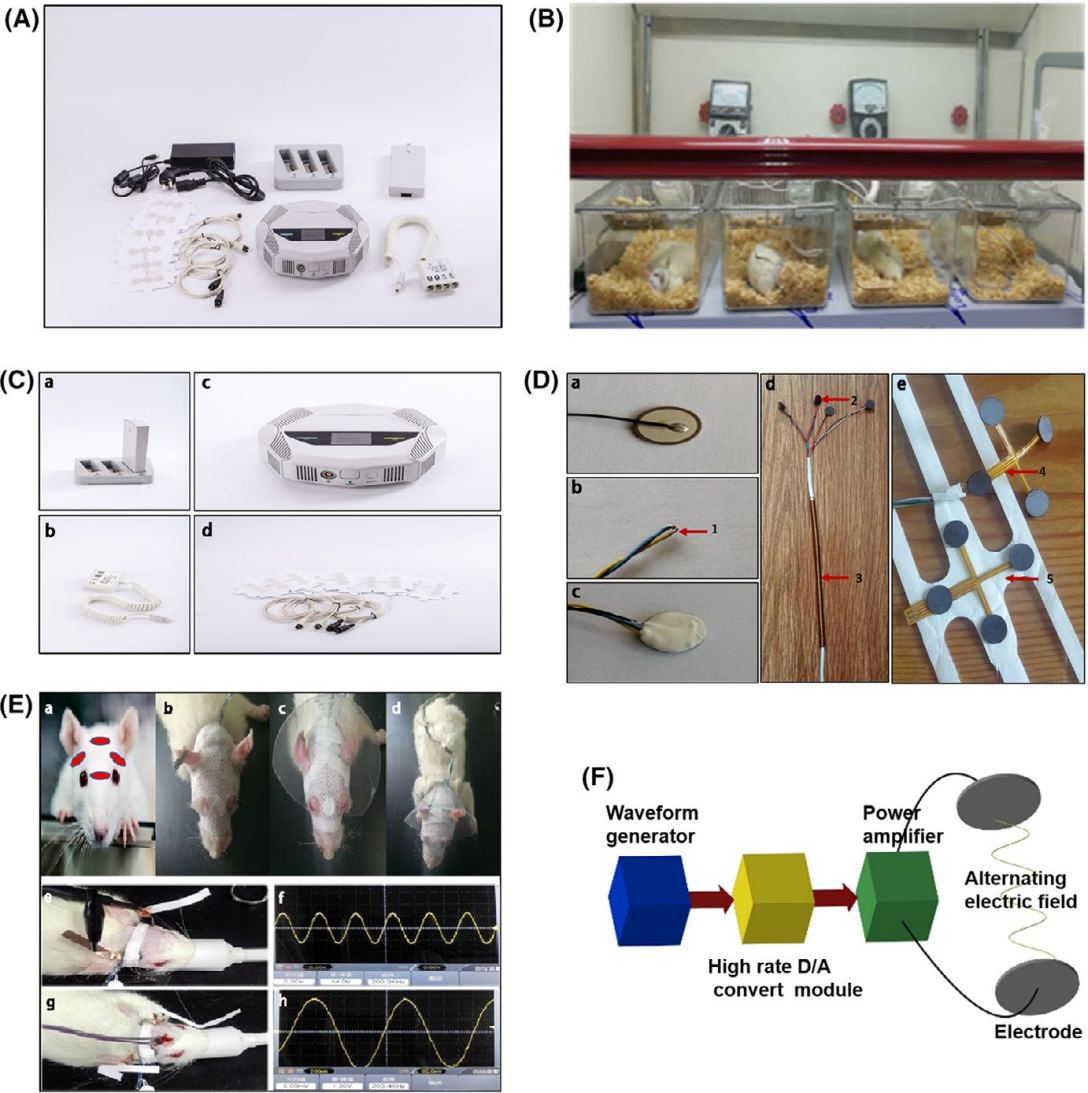
Tumor Electric Field Treatment System and Operation. A, Tumor Electric Field Treatment System (TEFTS, ASCLU‐300). B, Instrument operation during experiments. C, Main components of TEFTS. a, Battery and charger; b, Junction box and connecting cables; c, the electric field generator; d, Electrodes. D, Electrodes and accessories. a, On the back of electrode; b, Thermistor and circuit; c, after installing thermistor and circuit; d, Two pairs of ceramic disk electrodes. Thermistors on the back of electrodes are connected to a temperature‐controlling device. The diameter of the disk electrode is 8 mm (dielectric constant *ε* ≈ 10 000, capacitance *c* ≈ 10 nF). Four electrodes are used as one treatment unit. The wire is coated with metal to prevent from gnawing; e, two positive‐negative pairs of electrodes connected by a flexible circuit board are arranged perpendicularly to each other. The surface of the electrode is covered with hydrogel, and the back is fixed on medical adhesive tapes (1. Thermistor; 2. Disk ceramic electrode; 3. Metal piping coated wires; 4. Flexible circuit board; 5. Medical adhesive tape). E, Installation of electrodes and measurement of electric fields. a, the red dots indicate the locations of four electrodes on the head of a rat; b, the electrodes are fixed on head by tapes; c, a head cover protects the electrode from being scratched by the rat with its forelegs; d, the electrodes and a head cover are fixed; e, f, the voltage measured from the scalp; g, h, the intracranial voltage measured. F, Schematic diagram showing the generation of alternating electric field. Waveform generator built in the field programmable gate array (FPGA) generates the digital signal of sine wave according to the preset parameters. The digital signal is converted into analog signal by a high‐speed discretionary access control (DAC) module, and then filtered. The analog signal is amplified by a power amplifier, outputs to the electrode plate affixed to the therapeutic target, and generates an alternating electric field inside the target

The electrodes of the instrument are made of high dielectric constant ceramic materials. The four electrodes have a capacity ≈ 10 nF and a diameter of 8 mm. Each electrode is connected to a working electric generator and a temperature‐sensing device (Figure 2A‐D). The average temperature of the electrode was maintained at 36.0‐36.5°C throughout the experiment. During the course of treatment, the head was covered by medical adhesive tape and heat generated by electrode; as a result, the temperature may be slightly higher than that of non‐treated rats. The temperature setting of similar experiments was 40.5‐41°C to prevent low‐temperature burns, so we reduced the temperature to 40°C. A sensor automatically alarms when the temperature exceeds 40°C and the equipment stops working to ensure that the experiment is carried out safely and effectively. The working principle is shown in Figure 2F. The duration of timing of TEFT treatment is based on our preliminary experiments and previous publications using similar products.

Rats were anesthetized with isoflurane inhalation. The electrodes were fixed in the following four areas: two in the middle of lines between an ear and an eye of the same side, one between two ears, and one between two eyes (Figure 2Ea). The wires were fixed. A transparent head cover was applied to each rat to prevent bite (Figure 2Eb‐d). The electrodes were replaced every 2 days. The TTField treatment (200 kHz) was applied continuously for more than 20 hours per day. The control rats were equipped with the same device without electric field.

### 2.5 Magnetic resonance imaging (MRI) and tumor volume measurement

Rats were anesthetized with 3% pentobarbital sodium (35 mg/kg, intraperitoneally) and positioned on an animal cradle with a stereotaxic head holder. Gadolinium diamine (0.6 mmol/kg) was injected through the tail vein. T1‐weighted images of the tumor were acquired by a Ingenia 3.0 T‐PHILIPS MRI device (at The Third Medical Center of The General Hospital of the PLA, Beijing, China). The length and width of tumor were measured by the Syngofastview DICOM imaging device. Tumor volume was calculated with the following formula: Tumor Volume = length × width^ 2^/2 (mm^3^).^11^

### 2.6 Immunohistochemistry

PFA‐fixed tissues were paraffin embedded and sectioned into serial slices of 5 μm thickness. After permeabilization, antigen retrieval and blocking, the brain slices were incubated with primary antibody CD31 (Abcam, 1:1000) and cleaved caspase 3 (Abcam, 1:1000) diluted with 1% goat serum (Vector Laboratories) in PBS overnight. The slices were then incubated in the secondary antibody for 1 hour and stained with ABC HRP kit (Vectastain). After washing, the bound complex was visualized by incubating with DAB (3,3‐diaminobenzidine) and urea‐hydrogen peroxide. Images were obtained by Nikon–Eclipse Ti and Leica Aperio Versa 200. Image analysis was performed using the Image J 1.51j8 to calculate the positive stained areas and to count the number of positive cells.

### 2.7 Statistical analysis

GraphPad Prism 8.0 was used for statistical analyses. The Student's *t* test was used for comparison of two groups for continuous variables with normal distribution. The Mann‐Whitney *U* rank sum test was used for continuous variables with non‐normal distribution. The differences in means among multiple groups were analyzed using one‐way or two‐way analysis of variance (ANOVA) followed by the post hoc Bonferroni test for data with normal distribution and using one‐way ANOVA on ranks followed by the post hoc Dunn's test for data did not exhibit normal distribution. Survival was estimated using the Kaplan‐Meier method, and the survival curve was compared by log‐rank test. The difference was considered statistically significant when *P* < .05.

## 3 RESULTS

### 3.1 Measurement of intracranial field strength in rats

The distance between two probes was 5.04 mm. Two probes were inserted 8.00 mm below the skull to test the signals in between. The probes were insulated in the middle. One end (1.00 mm long) was exposed to detect the electrical signal. The other end was connected to the oscilloscope to observe the voltage difference and waveform of the signal (Figure 2Ee‐h). The voltage between the two needles (V) was 1.2Vp‐p. The frequency was 200 kHz. The sine wave and the interval time was 1 second. The head voltage of a rat was measured according to the formula Em = U/distance (1.2/0.504 ≈ 2.38Vpp/cm). This value was converted to a valid value according to the formula E = Em/2/√2 (2.38/2/√2 = 0.84V/cm). Therefore, the output parameter of the equipment was 18.0 Vp‐p, and the electrode remained 14.0 Vp‐p on the scalp of rats. The valid value was about 0.84 V/cm in the brain.

### 3.2 Effect of TEFTS on overall health and survival rate of glioma‐bearing SD rats

**FIGURE 3:**
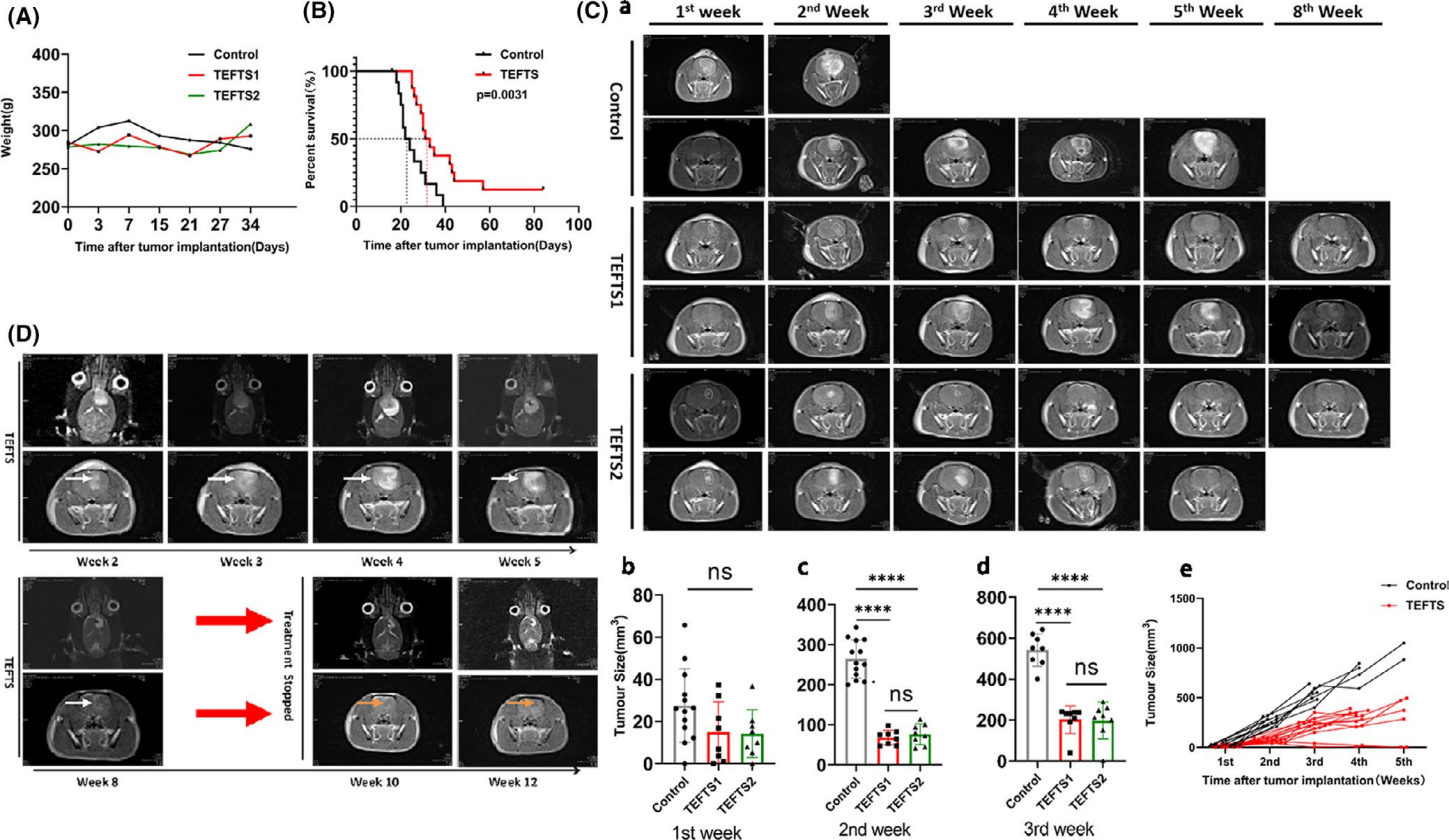
Effect of TEFTS on glioma growth in rats. A, Body weight. B, Survival curve. C, Tumor growth over time was monitored by MRI. Representative MRI images of TEFTS‐treated rats and non‐treated control rats from 1st to 8th weeks after tumor transplantation (a). Continuous TEFTS treatment significantly reduced tumor volume at 2nd and 3rd week after tumor inoculation (b‐d). Changes of tumor volume over time in individual rat were displayed in (e). D, Continuous TEFTS treatment for 8 wk. In the first 4 wk, the tumor volume gradually increased. The tumor volume was significantly reduced from the 5th week to the 8th week. After stopping the treatment, no rebound was observed at the 10th and 12th week. The white arrows show the tumor, orange arrows indicate the area after the tumor shrinks/disappears. *P < .05；** P < .01; ***P < .001; ****P < 0001

The overall health of the experimental animals was assessed daily after tumor cell inoculation. The rats in control or TEFTS treatment groups maintained their normal appetite and responded normally to the surroundings on day 2. With tumor growing, the rats in the control group exhibited rough hair coat, slow movement or motionlessness, reduced food consumption and fell over to the left when they moved. Blood stains around mouths, noses, and eyes were observed on most control rats without TEFTS treatment. The rats with TEFTS treatment showed improved general conditions. The body weights remained comparable from 0 to 27 days after tumor transplantation among the glioma‐bearing rats treated with TEFTS starting on either 1st (TEFTS1) or 3rd (TEFTS2) day after transplantation and those without TEFTS treatment (Figure 3A). Symptoms of epileptic seizures such as limb convulsions were not observed in TEFTS‐treated rats. The mortality of tumor‐bearing rats significantly decreased in TEFTS‐treated rats (Figure 3B, *P* = .0031). The average survival time for control rats was 24.77 ± 7.08 days, compared to 40.31 ± 19.11 days for the group treated by TEFTS.

### 3.3 Effect of TEFTS on tumor growth

MRI was applied to measure tumor size over time. As shown in Figure 3Ca,e, tumor volume increased significantly from 2nd to 5th week in control rats. In contrast, TEFTS treatment markedly slowed down the growth of glioma and even reduced tumor size in some animals. There was no significant difference in tumor volume between the TEFTS‐treated group and the control group during the first week (Figure 3Cb, *P* > .05). However, significant differences in tumor size were observed in the 2nd and 3rd week (Figure 3Cc,d, *P* < .0001). The two TEFTS treatment groups showed no difference in tumor volume (*P* > .05, Figure 3Cc,d). A longitudinal MRI scanning in one TEFTS‐treated rat showed that in the first 4 weeks, the tumor volume gradually increased with cystic degeneration (Figure 3D). The midline structure was displaced, and the contralateral brain tissue was compressed. The tumor volume was significantly reduced from the 5th week to the 8th week. The cessation of TEFTS treatment did not rebound tumor growth in the 10th and 12th week (Figure 3D).

**FIGURE 4:**
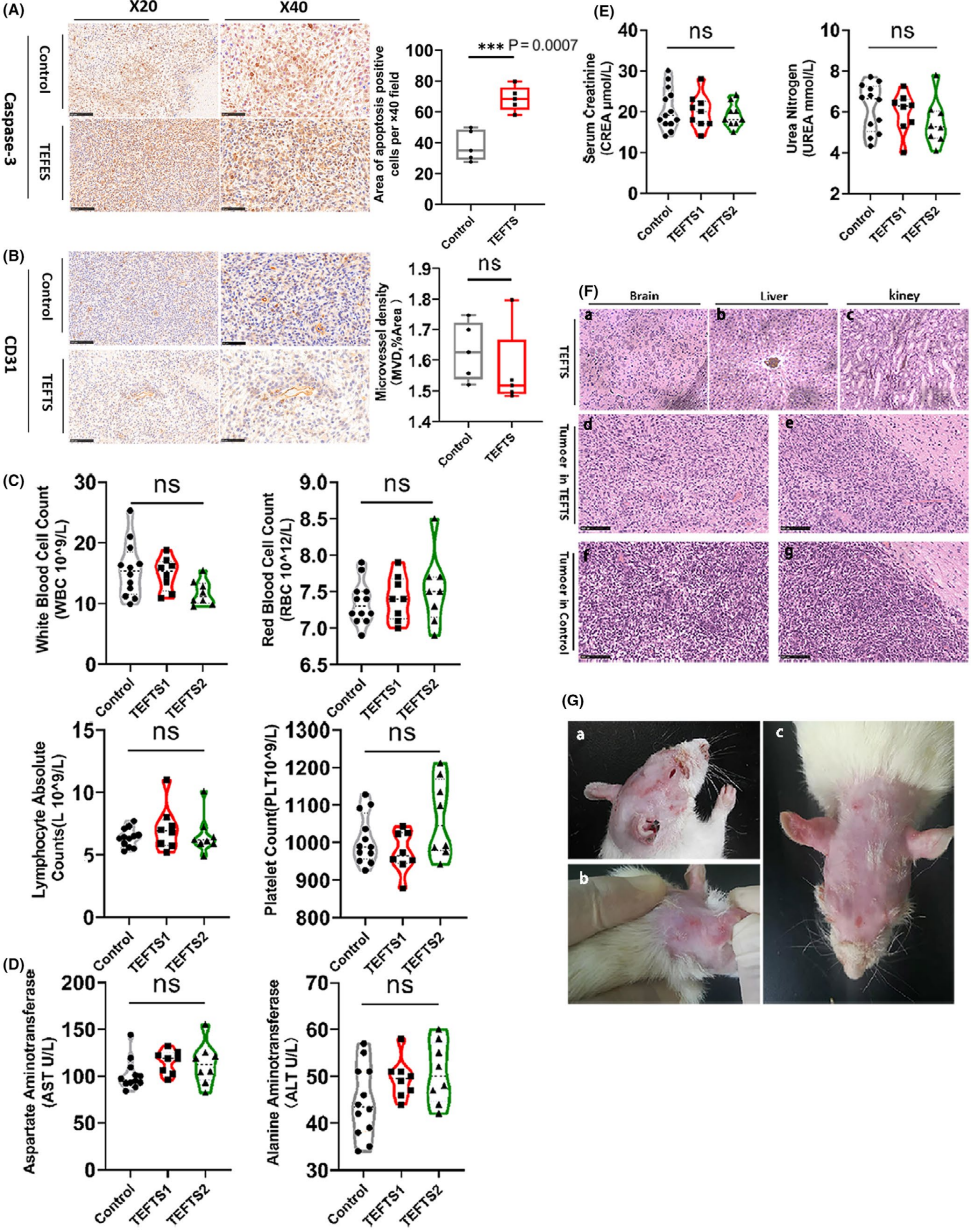
Safety of the TEFTS. A, Immunohistochemical staining of cleaved caspase‐3. The number of positive cells was counted under microscope (40×). B, Immunohistochemical staining of CD31. The area of positive staining was quantified. C‐E, Blood routine examination in each group on the 15th day of treatment. C, Blood cell counts; (D) alanine aminotransferase (ALT) and aspartate aminotransferase (AST) levels; (E), Urea nitrogen and serum creatinine levels among the three groups. F, Immunohistochemical staining of brain, liver, and kidney slices collected from the rats in different groups. G, Mild contact dermatitis on the skin where the electrode is attached. a,b, Mild rash in different parts of the electrode contacted areas; c, One week after the use of corticosteroids, the rash obviously has disappeared. *P < .05；**P < 01; ***P < .001; ****P < .0001. Scale bar = 100 μm

Furthermore, immunohistochemical staining confirmed an increased expression of caspase 3, a marker of cell apoptosis, within the tumor mass in TEFTS‐treated group compared to the control rats (Figure 4A, *P* = .0007). However, CD31 staining showed no significant difference between TEFTS‐treated rats and non‐treated controls (Figure 4B, *P* > .05).

### 3.4 Safety of TEFTS

The impacts of TEFTS on the functions of vital organs were evaluated 15 days after treatment. TEFTS treatment showed no effect on red blood cell count, white blood cell count, lymphocyte count, or platelet count (Figure 4C). In addition, TEFTS‐treated rats and control rats exhibited similar levels of serum glutamic oxaloacetic transaminase (AST, Figure 4D), alanine aminotransferase (ALT, Figure 4D), urea nitrogen (Figure 4E), and serum creatinine (Figure 4E), suggesting that TEFTS treatment did not impair liver or kidney functions.

HE staining was then performed in the brain, liver, and kidney slices (Figure 4Fa‐c) on day 20 of TEFTS treatment and revealed no differences between different treatment groups. In contrast, the HE staining demonstrated marked differences in tumor mass between TEFTS‐treated rats and control rats. Tumors in control rats exhibited hypercellular areas, in which striking tumor cellular pleomorphism, mitotic figures, invasive growth, and microangiogenesis were observed (Figure 4Ff,g). The tumor mass in TEFTS‐treated rats showed less cells and pathological mitotic figures, suggesting that the electric fields treatment had inhibited the growth of glioma cells (Figure 4Fd,e).

Local skin rash around the electrode fixation was the earliest adverse reaction observed (Figure 4G). The rash gradually disappeared with the use of corticosteroids.

## 4 DISCUSSION

As alternating electric fields of intermediate frequency and low intensity, the TTFields have been reported to slow down the growth of tumor cells while have no obvious bioeffects on normal cells.^11^ Our self‐designed tumor electric field therapeutic instrument is superior than other similar products in that it generates TTFields with random sequential electric field direction and provides adjustable frequency and intensity.

Precise spatial and temporal alignment of polarizable subcellular structures at different stages of cell division is necessary for successful mitosis. TTFields are known to interfere with microtubule formation and cause physical damages to cells, both of which rely on the relative direction of the mitotic axis and the field vector. However, due to the random orientation of the mitotic axis during cytokinesis, only a few mitotic cells are affected by TTField applied in a specific single direction. It is reported that increasing the direction of electric field from 1 to 3 significantly improved the anti‐proliferation effect of TTFields both in vitro and in vivo.^11^ Accordingly, our instrument with random sequential electric field direction can apply electric field to the rapidly proliferating tumor cells from all directions in order to achieve the best curative effects. Due to the small size of rat head, four electrodes were used in the current experiment to generate two electric fields perpendicular to each other.

The inhibitory effective of electric field therapy to the proliferation of various tumor cells depends on its frequencies and intensities.^11^ One advantage for our TEFTS instrument is that the intensity and frequency of generated electric field can be easily adjusted according to different requirements. Glioblastoma is a malignant tumor with pleomorphic cells of different volumes. Previous studies showed that the TTFields of specific frequency and strength may target a particular size of tumor cells. Moreover, tumor cells may change their size to escape from the TTFields.^11^, ^12^ Therefore, the optimal TTFields parameters need to be tuned based on the changes in cell size. Indeed, a study using two different human glioblastoma lines revealed cell line‐specific responses to TTFields.^8^ In addition, the application of TTFields may delay DNA damage repair following radiation treatment, leading to the emergence of therapeutic resistance.^8^, ^13^ All these conditions suggest that the therapeutic efficacy of TEFTS may vary between patients and in different stages of tumor, which necessitates the need to adjust the intensity and frequency of TTFields during the course of treatment. In the current study, we tested the effect of our TEFTS instrument in only one glioma cell line. Future study using other cell lines will confirm whether the capacity of our instrument to adjust the intensity and frequency of electric field will widen the range of its application to different subtypes of glioblastoma.

Our data showed that the TEFTS initiated at 1 or 3 days after tumor transplantation had similar efficacy to inhibit tumor growth. However, when we delayed the TEFTS treatment till 7 days after transplantation, it failed to reduce tumor volume or improve overall survival time (data not shown). Such unsuccessful delayed treatment might be due to several reasons. First, the heterogeneity of malignant tumor^14^, ^15^ in late stage results in uneven electric fields and reduces effective voltage within the tumor. Second, the tumor cells in late phase are less sensitive to TEFTS treatment compared to early stage tumor cells due to alterations in size and proliferation activity. Third, long‐term continuous treatment is required for TEFTS treatment to control the growth of tumor cells before their fatal impacts on animals occur. Nonetheless, with the development of approaches for early diagnosis of glioblastoma, the TEFTS treatment initiated in early stage is expected to slow down the progress of devastating glioblastoma.

Neovascularization is indispensable in the process of tumor growth, invasion, and metastasis.^16^, ^17^, ^18^A number of molecules and specific molecular alterations have been found to play a role in angiogenesis regulation within brain glioma that, potentially, may serve as molecular biomarkers and/or therapeutic targets.^19^, ^20^, ^21^, ^22^, ^23^ Several studies showed that TTFields could directly impact blood circulation within tumor through inhibiting blood vessel growths.^24^ Chen et al found that exposure to TTFields down‐regulated the expression of CD34, an important marker of tumor vasculature, in malignant melanoma cell lines.^25^ Nuccitelli et al also reported that electric fields reduced tumor blood flow and initial melanoma cell pyknosis, resulting in the reduction of tumor volume.^26^ In the current study, to determine whether TEFTS inhibits tumor growth by targeting microvessels, we examined CD31 expression in glioma tissues using immunohistochemistry, but failed to find a significant difference in CD31 expression levels between TEFTS‐treated group and control group. Our results suggest that using the current therapeutic parameters (intensity and frequency), TEFTS does not directly inhibit angiogenesis and the density of microvessels in glioma tissues. Thus, a combination approach with both TEFTS and anti‐angiogenic therapy, such as regorafenib,^27^ should be considered for our future studies. Such combo approach may be particularly relevant for glioma therapy when TEFTS treatment is to be delayed and TEFTS alone may not be effective. We further suggest that detection of the established molecular or imaging biomarkers for glioma before and after TEFTS treatment may also help evaluate efficacy and make prognostic estimations.

The parameters for Novocure TTF‐100A, a commercially available tumor electric field therapeutic instrument, to inhibit glioblastoma in vivo and in vitro are 200 kHz frequency and 0.7‐1.4 V/cm intensity.^11^ In our in vivo experiment, the parameters for TEFTS (ASCLU‐300) were set at similar parameters of 200 kHz frequency and 0.84 V/cm intensity. The TEFTS treatment with ASCLU‐300 prominently prolonged the survival time from 24.77 ± 7.08 to 40.31 ± 19.11 days. It is noted that different parameters were employed in Kirson's research using a melanoma lung metastasis model.^28^ In Kirson's study, the experimental treatment lasted for 7 days, with lower frequency of 100 kHz, and average effective intensity in the lung at 1.8‐2 V/cm. Such differences may suggest tumor cell‐specific sensitivities to electric fields therapy.

Chemotherapy or radiotherapy is conventional adjuvant therapy after malignant tumor resection. However, the long‐term application of these therapies is often associated with systemic toxicity and the development of single‐drug resistance or multidrug resistance (MDR).^29^, ^30^ The electric fields therapy has obvious advantages compared with chemotherapies or radiotherapies, including higher CNS cell accessibility, fewer systemic side effects, and relatively rare resistance after long‐term application. Indeed, we found that long‐term electric field therapy had no adverse effect on important tissues or organs, including blood, liver, and kidney. We did notice that 31.25% of the rats developed mild contact dermatitis in the electrode‐contacting area during the treatment. This was due to bad local air ventilation after long‐term use of conductive adhesives and tapes, the heat produced by the electrode transducers and increased skin secretions. The contact dermatitis can be prevented or cured by replacing electrodes regularly and wiping the skin with corticosteroids.

Therapeutic resistance, although occurred less frequently than chemotherapy, is still a concern for electric fields therapy. As mentioned above, the TEFTS therapy can induce genetic changes and mutations of the DNA repair mechanism in tumors.^31^ In addition, the size of glioma cells, the heterogeneity of local tissue, and the uneven field strength within the tumors are all related to electric field therapy resistance. Interestingly, it is reported in ovarian carcinoma cells that the resistance to electric fields therapy might be reversed by reducing the frequency of electric fields.^32^ Hopefully, with its capacity to provide adjustable frequency and intensity, the ASCLU‐300 instrument described in the current report can at least partially overcome the resistance to TEFTS therapy.

## 5 CONCLUSIONS

The TEFTS we developed inhibits tumor growth and elongates overall survival in an in vivo model of glioblastoma. This instrument can be effectively and safely used for glioma treatment. Our understanding to the electric field therapy and the mechanisms of action is still limited. Therefore, longitudinal imaging during the course of treatment is necessary to further optimize the parameters of TEFTS and adjust the therapeutic regimen. Although the electric fields therapy cannot replace traditional treatments yet so far, it has raised hopes to patients with glioma. With the development of precision medicine, electric fields therapy may provide strong powers for human beings to battle glioma and other solid tumors.

## CONFLICT OF INTEREST

The authors declare no conflict of interest.
